# Gold nanoparticles combat enveloped RNA virus by affecting organelle dynamics

**DOI:** 10.1038/s41392-023-01562-w

**Published:** 2023-08-02

**Authors:** Fangzhou Li, Qianqian Huang, Ziran Zhou, Qiongge Guan, Fei Ye, Baoying Huang, Weisheng Guo, Xing-Jie Liang

**Affiliations:** 1 CAS Key Laboratory for Biomedical Effects of Nanomaterials and Nanosafety, CAS Center for Excellence in Nanoscience, National Center for Nanoscience and Technology of China, No. 11, First North Road, Zhongguancun, 100190 Beijing, P. R. China; 2 University of Chinese Academy of Sciences, 100049 Beijing, P. R. China; 3 MHC Key Laboratory of Biosafety, National Institute for Viral Disease Control and Prevention, China CDC, Beijing, China; 4 Department of Minimally Invasive Interventional Radiology, the State Key Laboratory of Respiratory Disease, School of Biomedical Engineering & The Second Affiliated Hospital, Guangzhou Medical University, 510260 Guangzhou, P. R. China

**Keywords:** Nanobiotechnology, Cell biology, Nanobiotechnology

## Abstract

Enveloped RNA viruses are a group of viruses with an outer membrane derived from a host cell and a genome consisting of ribonucleic acid (RNA). These viruses rely on host cell machinery and organelles to replicate and assemble new virus particles. However, the interaction between viruses and host organelles may be disrupted by nanomaterials, such as gold nanoparticles (AuNPs) with unique physical and chemical properties. In this study, we investigated the effects of AuNPs with different surface charge properties on the subcellular structure and function of mammalian cells, and their effects on two representative enveloped RNA viruses: lentivirus and human coronavirus OC43 (HCoV- OC43) antiviral potential. By comparing the subcellular effects of AuNPs with different surface charge properties, we found that treatment with AuNPs with positive surface charges induced more significant disruption of subcellular structures than neutrally charged AuNPs and negatively charged AuNPs, mainly manifested in lysosomes and Cytoskeletal disorders. The antiviral effect of the surface positively charged AuNPs was further evaluated using lentivirus and HCoV-OC43. The results showed that AuNPs had a significant inhibitory effect on both lentivirus and HCoV-OC43 without obvious side effects. In conclusion, our study provides insights into the mechanism of action and biocompatibility of AuNP in biological systems, while supporting the potential of targeting organelle dynamics against enveloped RNA viruses.

## Introduction

RNA-enveloped viruses are a group of viruses with a single-stranded or double-stranded RNA genome and a lipid membrane from the host cell. They are responsible for various human diseases, such as influenza, SARS-CoV-2, hepatitis C, Ebola, and rabies,^1,2^ ranging from mild respiratory infections to severe hemorrhagic fevers and neurological disorders.^3^ RNA-enveloped viruses have high mutation rates, which make them more adaptable to changing environments and immune responses, but also more prone to error catastrophe.^3,4^ There are different antiviral strategies for RNA-enveloped viruses, such as vaccines, monoclonal antibodies, peptide-based inhibitors, chemical compounds, CRISPR-based approaches, etc.^5^ However, additional antiviral strategies are needed because RNA-enveloped viruses are constantly evolving and adapting, which can reduce the effectiveness of existing vaccines and antivirals. Moreover, some RNA-enveloped viruses have no approved or available antiviral therapies, which poses a serious threat to public health and security. Therefore, new antiviral targets, mechanisms, and modalities that can prevent or treat infections by RNA-enveloped viruses must be explored and exploited.

SARS-CoV-2 has currently created a huge demand for antiviral strategies, which relies on an in-depth understanding of biological mechanisms. Different stages of viral infection, such as viral invasion, RNA replication, or viral clearance, are targeted by ongoing clinical trials.^6^ Another potential target for antiviral therapy is the process of virus assembly and transport, which depends largely on organelle dynamics.^3,7,8^ Organelle dynamics refers to the processes of organelle biogenesis, movement, fusion, fission, and degradation that regulate the shape, size, number, and distribution of organelles within a cell.^9,10^ The dynamics and interactions of organelles in relation to viral infection are complex and diverse. For example, viruses enter host cells through different pathways that involve various organelles, such as plasma membrane, endosomes, lysosomes, or Golgi apparatus.^11^ Viruses also hijack the cytoskeleton and motor proteins to facilitate their intracellular transport and delivery to the site of replication. For instance, microtubules and dynein mediate the retrograde transport of viruses from the plasma membrane to the nucleus or other organelles.^12^ Therefore, targeting subcellular structural homeostasis could be a promising antiviral strategy. However, conventional strategies such as small molecule drugs are challenging in inducing reversible regulation of subcellular homeostasis. In contrast, biologically inert gold nanoparticles offer advantages.^13^

AuNPs are nanoscale particles of gold that have unique properties for various clinical applications, such as drug delivery, photothermal therapy, contrast enhancement and gene transfection.^14–20^ AuNPs have a large specific surface area and are easily modified, which enables them to bond with drugs chemically or nonchemically and form a drug delivery nanosystem. This can increase drug solubility, reduce cytotoxicity and improve drug stability. Therefore, AuNPs have been widely used in various drug delivery applications. Moreover, AuNPs are important model particles for nano-biological effects and are widely used to explore the mechanisms of nano-biological effects, such as the enhanced permeability and retention (EPR) effect and subcellular effects of nanostructures.^13,21^ Appropriate treatment with gold particles is widely considered to allow the AuNPs to be taken up by the cells without affecting their viability,^13,22,23^ thereby, we hypothesize that AuNPs may play a positive role in inhibiting viral replication by induing perturbation of subcellular structures. Furthermore, the surface charge properties of nanoparticles are an important feature that affects their biological effects. The surface charge properties can change the interaction mode between nanoparticles and biological systems, such as proteins, cells, tissues, and organs. A deeper understanding of the biological effects of different surface charge properties can help us modulate the interaction patterns between nanoparticles and biological systems, and thus control the fate of nanoparticles in vivo. Therefore, the study of the biological effects of the surface charge properties of nanoparticles is essential to advance their biomedical applications.

Targeting organelle dynamics with AuNPs could potentially interfere with viral infection and replication. But this strategy would require careful evaluation of the antiviral efficiency and the possible side effects on normal cellular functions and homeostasis. In this study, we compared the subcellular effects of 50-nm AuNPs with different surface charge properties for the potential of antiviral activity. The results showed that surface positively charged AuNPs had a greater effect on lysosomal behaviors, causing more significant lysosomal swelling, alkalinization, and inhibition of motility. Consistently, positively charged AuNPs induced disorders of organelle dynamics, here the phenomenon was manifested with a disruption of the cytoskeleton. To confirm the antiviral activity of the AuNPs, we infected the cells with lentivirus or HCoV-OC43 and measured the viral load and infectivity after the treatment with different concentrations of positively charged AuNPs. We found that positively charged AuNPs significantly reduced the viral load and infectivity of both lentivirus and HCoV-OC43. Importantly, we did not observe any obvious cytotoxicity or apoptosis in the cells treated with positively charged AuNPs, indicating that they were safe and biocompatible. Collectively, the surface positively charged AuNPs possessed antiviral activity against lentivirus and HCoV-OC43 by disrupting the lysosomal and cytoskeletal dynamics of the host cells without causing any obvious side effects.

## Results

### AuNPs with different surface charge properties accumulated into and enlarged lysosomes

**Fig. 1:**
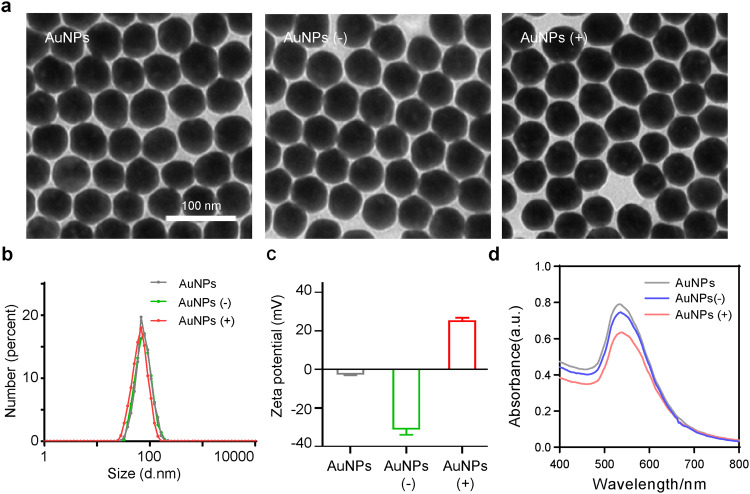
Characterization of AuNPs with different surface charge properties. **a** TEM images of AuNPs (scale bar, 100 nm). **b** Hydrodynamic diameters of the respective AuNPs measured by DLS. **c** Measured zeta potential of AuNPs. **d** UV–Vis absorption spectrums of AuNPs

**Fig. 2:**
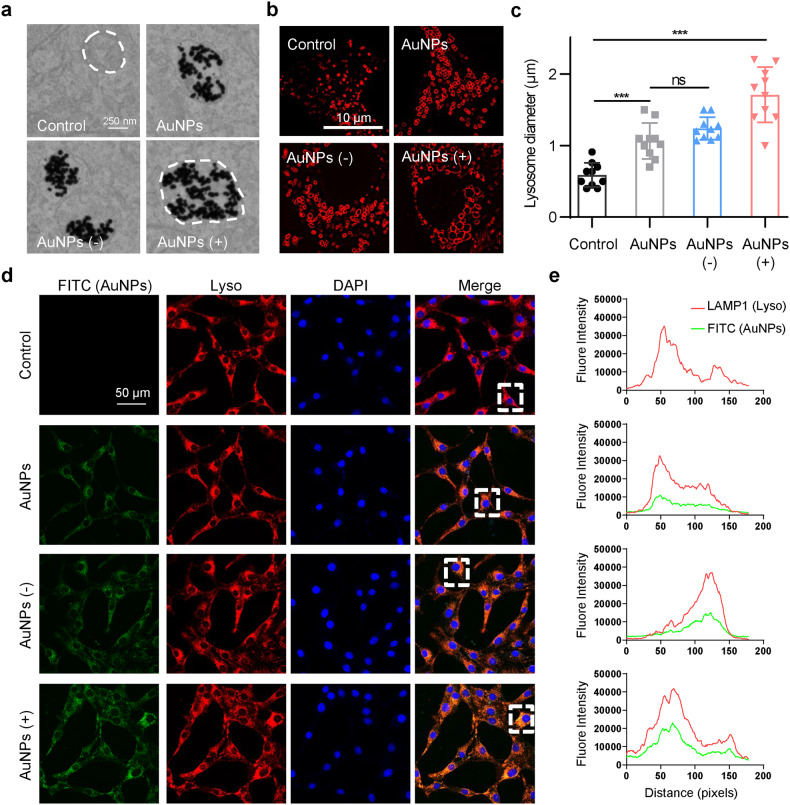
AuNPs accumulation leads to lysosome enlargement. **a** TEM images of NRK cells untreated or treated with AuNPs (scale bar, 5 μm). The right images are the enlarged views of the lysosome in the circled area (scale bar, 250 nm). **b** Staining pictures of lysosomes within living cells. NRK cells were incubated for 24 h with plain medium (control) or with 1 nM AuNPs (scale bar, 10 μm). **c** Statistical values of corresponding lysosome diameters. Data are represented as mean ± SEM, *n* = 10 per group. **d** Confocal pictures of lysosome marked with LAMP1 (red) and FITC-labeled AuNPs (green) (scale bar, 50 μm). **e** Co-localization profiles of lysosome and FITC-labeled AuNPs were shown on the right and were analyzed with the randomly circled white box in the merge images

First, three types of 50-nm colloidal AuNPs with different surface charged properties were prepared and characterized to investigate and compare their subcellular effects (Fig. 1a–d and Supplementary Table 1). Normal rat kidney (NRK) cells were treated with 1 nM AuNPs for 24 h. None of the three types of AuNPs had any effect on cell viability at this concentration (Supplementary Fig. 1). The TEM images showed that all the three types of AuNPs accumulated in lysosomes (Fig. 2a). Both the TEM and immunofluorescence images showed that AuNPs treatment led to an enlargement of the lysosomes (Fig. 2a–c). The lysosomes from the control group showed a typical punctate structure, and AuNPs with different surface charge properties caused different degrees of lysosome enlargement, wherein positively charged AuNPs caused greater lysosomal swelling than the other groups (Fig. 2a–c). The signals co-localization of FITC-AuNPs with RFP-tagged lysosomal-associated protein 1 (LAMP1) further supported the results (Fig. 2d, e).

### AuNPs with different surface charge properties treatment leaded to disturbed lysosomal behavior

**Fig. 3:**
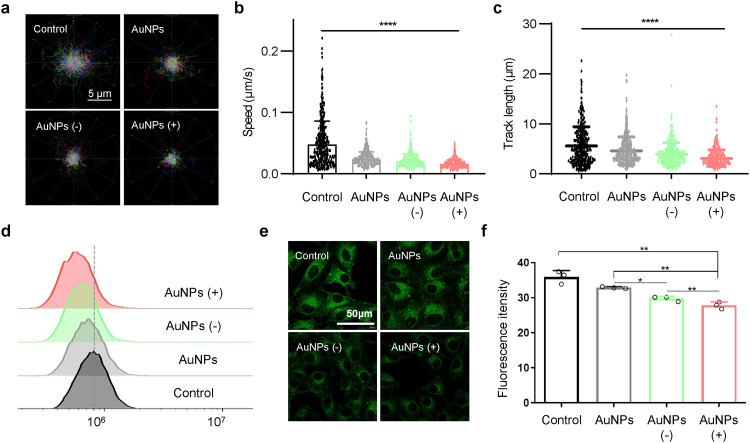
Disorders of lysosomal behavior induced by AuNPs. **a** The corresponding trajectories of lysosome mobility measured over 5 min with or without AuNPs. **b** The average speed of lysosome movement. Data are represented as mean ± SEM, *n* ≥ 450 per group. **c** Scatter plots of the track length of lysosome movement. Data are represented as mean ± SEM, *n* ≥ 450 per group. **d** Flow cytometry analysis of lysosome pH after AuNPs treatment. **e** Representative CLSM pictures of cells treated with AuNPs, and then stained with LysoSensor Green DND-189 (scale bar, 50 μm). **f** Statistical analysis of the relative fluorescence intensity of LysoSensor per cell after AuNPs treatment

After 24 h of AuNPs treatment, we recorded lysosomal motility using structured-illumination microscopy (SIM). Consistently, the results showed that the inhibition of lysosomal motility by positively charged AuNPs was more pronounced than in the other groups (Fig. 3a–c and Supplementary Videos 1–4). We then investigated the impact of AuNPs treatments on lysosomal pH. The signal analysis of the treated cells stained with lysoSensor™ Green DND-189 revealed that all three AuNPs caused lysosomal alkalinization, and the lysosomal pH was highest in cells treated with positively charged AuNPs, compared to the other cells (Fig. 3d–f). Collectively, positively charged AuNPs brought the most significant effect on lysosomal behaviors without obvious side effects on cell viability.

### Surface positively charged AuNPs affected organelle dynamics

**Fig. 4:**
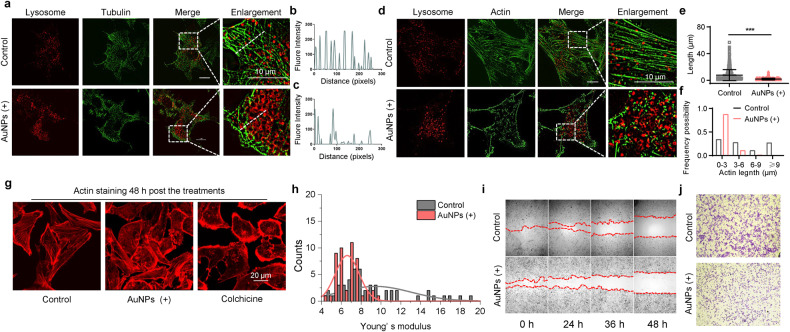
AuNPs (+) treatment induced organelle dynamics disorders. **a** High-resolution SIM pictures of co-label of lysosome (red) and tubulin (green) with or without AuNPs treatment. The last column: close-up of the enlarged pictures (scale bar, 10 μm). **b**, **c** The fluorescence distribution of tubulin at the white dashed line. **d** High-resolution SIM pictures of co-label of lysosome (red) and F-actin (green) with or without AuNPs treatment. The last column: close-up of the enlarged picture (scale bar, 10 μm). **e**, **f** Statistic analysis of microfilament lengths. The upper diagram shows the microfilament length (**e**), the lower diagram is microfilament length distribution (**f**). Data are represented as mean ± SEM, *n* ≥ 660 per group. **g** After treatment of positively charged AuNPs, NRK cells were incubated with fresh medium for another 48 h. Cytoskeleton (Actin) confocal analysis using NRK cells untreated (control) or treated with positively charged AuNPs (1 nM) or colchicine (5 μM). **h** Histogram of Young’s modulus distribution of untreated (control) and AuNPs-treated cells, the analyzed cell number are 15 in each group. The inserted curves are Gaussian fitting of Young’s modulus distribution. **i** Representative wound-healing assay pictures of cells treated with or without AuNPs. Pictures of cells were taken at 0, 24, 36, and 48 h to analyze the dynamics of wound closure, and red dotted line indicates the wound. **j** Representative images of transwell invasion assays showing decreased migration abilities of cells treated with AuNPs for 24 h

Surface positively charged AuNPs were further investigated for the antiviral potential by causing subcellular structural disorders. Cellular cytoskeleton plays an important role in maintaining subcellular structural homeostasis. The disruption of lysosomes can have an impact on cytoskeleton through the direct interaction between the cytoskeleton and lysosomes.^23,24^ The labeled lysosomes and microtubules were imaged using high-resolution microscopy. In the control group, the distribution of microtubules was homogeneous and ordered, accompanied by smaller lysosomes. After treatment with surface positively charged AuNPs, the distribution and integrity of microtubules were significantly disturbed (Fig. 4a). Analysis of the fluorescence signal distribution of microtubules further supported the effect of AuNPs on microtubules (Fig. 4b, c). Microfilaments are helically polymerized actin molecules, another key part of the cytoskeleton. Similar to microtubules, the integrity of microfilaments was significantly compromised by AuNPs treatment (Fig. 4d), as evidenced by a decrease in the average length of microfilaments (Fig. 4e) and an increase in the number of short microfilaments (Fig. 4f). Furthermore, we extended the observation of the cytoskeleton to determine whether the cytoskeletal perturbation is reversible in response to positively charged AuNPs treatment. Compared to colchicine treatment, a cytoskeleton-destroying small molecule, the cytoskeletal homeostasis after positively charged AuNPs treatment showed a trend towards restoration after 48 h, suggesting positively charged AuNPs caused mild effects on organelle dynamics (Fig. 4g). As disruption of the cytoskeleton leads to a decrease in cell stiffness,^25,26^ atomic force microscopy (AFM) indentation method was used to measure the Young’s modulus of NRK cells after AuNPs treatment (Supplementary Fig. 2). Thousands of raw force curves were obtained from multiple cells, and Young’s modulus values were calculated from a Gaussian fit to the Young’s modulus distribution (Fig. 4h). The average Young’s modulus value for the AuNPs-treated cells were approximately 6.59 ± 1.19 kPa, which was significantly lower than that of the control cells (9.43 ± 3.12 kPa). The integrity of the cytoskeleton is a guarantee of cell migrate. Serum-free cell migration assays (Fig. 4i) and transwell assays (Fig. 4j) were used to evaluate the motility of the cells. The results showed that positively charged AuNPs treatment significantly slowed the movement of the cells. The inhibition of cell motility by positively charged AuNPs treatment was further supported by high-content microscopy observations (Supplementary Fig. 3). Taken together, these data suggest that AuNPs treatment triggered disruption of subcellular structure, manifested here as a disruption of the cytoskeleton. We further evaluated the biosafety of the positively charged AuNPs used. The measurement results of the cell viability showed that AuNPs in the concentration range we used had no significant effect on NRK cell viability (Supplementary Fig. 4).

### Surface positively charged AuNPs inhibited two different enveloped RNA viruses

**Fig. 5:**
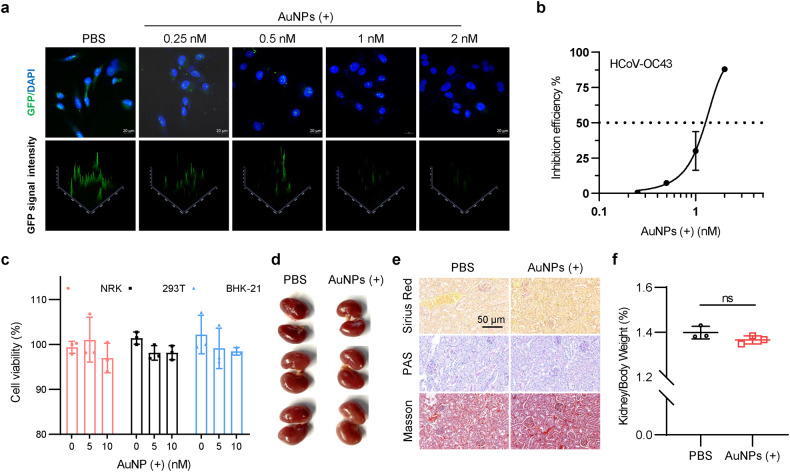
AuNPs (+) inhibited lentivirus and HCoV-OC43 without significant kidney side effect. **a** The virus-contained medium was collected for NRK cells infection, which were then prepared for GFP visualization (scale bar, 20 μm). **b** Different concentrations of AuNPs (+) were used to treat HCoV-OC43 infected BHK-21 cells. The inhibition efficiency was determined by RT-PCR. **c** Cell viability of the three different cell lines was assayed after the treatments with 0, 5 nM or 10 nM AuNPs (+). Data are represented as mean ± SEM, *n* = 3 per group. **d** Images of the excised kidneys on day 21. **e** Representative histological images of picrosirius red, PAS, and Masson’s trichrome staining in the kidneys (scale bar, 100 µm). **f** Kidney weight to body weight ratio. Data are represented as mean ± SEM; *n* = 3 per group

Lentivirus is a viral system based on the HIV-1 and is widely used in basic research, which we used to study the antiviral potential of AuNPs. The GFP reporter contained triple plasmid system (psPAX2-pMD2.G-pLL3.7) was used for 293T cell-based lentivirus production. As shown in Supplementary Fig. 5, AuNPs treatment hardly affected the expression of GFP reporter gene, suggesting that AuNPs did not affect the transcriptional-translational system of the host cells. And then, the medium containing virus was collected for NRK cells infection. The results showed that the medium from the control group resulted in strong GFP signal, whereas the medium from gold nanoparticle-treated groups produced relatively less GFP signal, while the signal intensity showed a dose-dependent relationship with the concentration of AuNPs (Fig. 5a). These results provided a preliminary indication of the advantages of gold nanoparticles in antiviral. Human coronaviruses (HCoVs) are enveloped RNA viruses that frequently cause widespread transmission. The antiviral effects of positively charged AuNPs against HCoV-OC43 were further investigated. The results showed that the positively charged AuNPs could significantly inhibit the infection of BHK-21 cells by HCoV-OC43, wherein the inhibition efficiency of 2 nM positively charged AuNPs reached more than 80% (Fig. 5b). Meanwhile, with the treatments of relatively higher concentrations of gold nanoparticles (2 nM and 5 nM), the cell viability of the three cell lines was not significantly impaired (Fig. 5c).

Kidney plays a key role in the filtration and elimination of nanoparticles from the blood.^9^ We tried to find out whether AuNPs could cause side effects to the kidney in vivo. The mice were intravenously injected with 50-nm AuNPs solution (90 nM, 100 μL/per 3 day/mouse) for 21 days. No abnormalities in kidney morphology were found in the AuNPs-treated group (Fig. 5d). The results were further supported by the picrosirius red, periodic-chiff (PAS), and Masson’s trichrome staining with the kidneys (Fig. 5e). The body weight and the ratio of kidney weight to body weight did not show significant changes in the AuNPs-treated group compared to the control group (Supplementary Fig. 6 and Fig. 5f).

## Discussion

In this study, we found that AuNPs could affect subcellular structural homeostasis by influencing organelle interaction networks. AuNPs accumulate in lysosomes, and positively charged AuNPs were found to cause the most significant disruption of lysosomal behaviors, such as enlargement, alkalinization and reduced motility. These changes affect the interaction between lysosomes and cytoskeleton, which then affect the integrity and distribution of microtubules and microfilaments. These effects can disrupt the subcellular structural homeostasis and impair the normal functions of the cell. The cellular substructural homeostasis was most affected by the surface positively charged AuNPs. This may be because positively charged NPs can interact more with the negatively charged cell membrane structures in various ways, compared to neutral and negatively charged AuNPs. For biomedical research, AuNPs has been shown to exhibit antiviral activity by some different mechanisms.^27,28^ For example, AuNPs were shown to bind to the glycoprotein spikes of herpes simplex virus type-1 (HSV-1) and block its interaction with cellular heparan sulfate proteoglycans (HSPGs), which are essential for viral infection.^27^ In our study, we used gold nanoparticles to interfere with organelle dynamics and organelle interactions, which resulted in the suppression of the virus. Although we maintain that targeting the subcellular structural homeostasis is a viable antiviral strategy, there is clearly a long way to go before this AuNPs-based strategy can be clinically applied. In addition, more detailed mechanistic studies and in vivo validation trials for viral inhibition with AuNPs should be conducted in the near future.

Organelle dynamics have been shown to be critical for virus infection. In addition to the lysosomes and cytoskeleton that we have focused on in this study, there are many organelles that play an important role in viral infestation of the host. For viral replication and assembly, viruses remodel the membranes of intracellular organelles to create viral replication complexes or factories, where they synthesize their genomes and protein. These organelles include endoplasmic reticulum (ER), mitochondria, peroxisomes, or autophagosomes.^29,30^ For example, viruses can use the ER-associated protein degradation (ERAD) pathway to degrade host proteins and evade immune responses.^31^ Viruses can also use the ER-Golgi intermediate compartment (ERGIC) or the trans-Golgi network (TGN) to acquire their lipid envelopes.^32^ For viral egress and release, viruses exit host cells through different mechanisms, such as budding, exocytosis, or cell lysis.^33,34^ These mechanisms involve various organelles, such as plasma membrane, endosomes, lysosomes, or multivesicular bodies (MVBs). For instance, viruses induce the formation of vesicular stomatitis virus glycoprotein (VSVG)-containing vesicles that fuse with the plasma membrane and release viral particles.^35^ Viruses also induce the formation of extracellular vesicles (EVs) that carry viral components and modulate intercellular communication.^36^ In addition, host cells would mount various responses to counteract viral infection, such as innate immunity, apoptosis, or autophagy,^37^ which also involve various organelles.

Of all these organelles, the dynamics of the ER, lysosomes, and mitochondria appear to be especially important for viral infection. The ER forms vesicles or tubules from its membrane that help with the synthesis, modification and assembly of viral proteins and particles.^30^ Viruses can induce ER stress and the unfolded protein response (UPR) to regulate ER dynamics and enhance viral replication.^38^ Lysosomes fuse with endosomes or autophagosomes to degrade viral particles or components and activate innate immune receptors. To avoid degradation and enhance infection, viruses can manipulate lysosomal dynamics in various ways. For instance, coronaviruses can reduce the acidity of lysosomes, which prevents them from destroying viral particles or components. This allows the viruses to escape from lysosomes intact and infect other cells.^39,40^ Influenza virus can inhibit the fusion of lysosomes with endosomes or autophagosomes, which delivers viral cargo to the lysosomal lumen. This enhances viral replication and suppresses innate immunity. Some viruses can also use lysosomal exocytosis, a process that normally removes cellular waste or releases signaling molecules, to exit cells without damaging the plasma membrane. For example, dengue virus can induce this process to spread to neighboring cells.^41^ Viruses can disrupt lysosomal dynamics to avoid degradation or immune signaling.^40^ Mitochondria undergo fusion and fission to balance their dynamics and produce antiviral molecules, such as interferon, ROS, apoptosis and autophagy. Viruses can alter mitochondrial dynamics to evade or inhibit these antiviral mechanisms.^42^ Altogether, organelles play a crucial role in both viral infection and the body’s defense against viral infection, and understanding the dynamics of these organelles and their interactions with viruses is crucial for developing antiviral strategies.

AuNPs have been developed for various biomedical applications, but they still encounter many challenges and barriers for their clinical use. One challenge is the stability of AuNPs in the physiological environment, which can be affected by salt, pH, temperature, proteins and enzymes. These factors can alter the size, shape, surface charge and optical properties of AuNPs, as well as their biodistribution, biocompatibility and antiviral activity. To improve the performance and safety of AuNPs, different functional ligands are usually added to modify their surface. However, functionalization can also change the physicochemical and optical properties of AuNPs, as well as their interactions with biological systems. Another challenge is the targeting of AuNPs to specific tissues or organs. AuNPs can easily enter different cells due to their nano size, but this also reduces their selectivity and specificity. To increase their affinity for certain receptors or biomarkers, targeting molecules such as antibodies, peptides or aptamers are often conjugated to AuNPs. However, targeting can also increase the complexity and cost of AuNPs synthesis and characterization. A third challenge is the toxicity and biocompatibility of AuNPs. Compared to other nanoparticles, AuNPs are generally considered to have low toxicity and high biocompatibility, but they can still cause adverse effects depending on various parameters, such as size, shape, surface charge, concentration, exposure time and route. AuNPs can interact with various biological components such as proteins, DNA, membranes and organelles, and induce oxidative stress, inflammation, apoptosis or genotoxicity. AuNPs can also accumulate in certain organs or tissues and cause long-term toxicity or clearance problems. Further research is needed to optimize these parameters and evaluate the safety and efficacy of AuNPs in animal models and clinical trials.

## Materials and methods

### AuNPs synthesis and characterization

In all, 50-nm colloidal AuNPs were prepared by the citric acid reduction method.^43^ Specifically, 100 mL of Milli-Q water was added to a three-necked flask with 1 mL of 1% HAuCl_4_. After heating the solution to boiling with vigorous stirring, 1 mL of 1% sodium citrate was rapidly injected and kept vigorously stirred to obtain 50 nm AuNPs. According to molar ratio of Au:S = 1:10,000, SH–PEG–OCH_3_, SH–PEG–COOH, SH–PEG–NH_2_ were added slowly into the solution and stirred for 8 h. After the reaction was completed, washed, and centrifuged the solution three times (5700 rpm for 10 min) to remove excess free PEG molecules to obtain neutral, positive, and negative charged AuNPs, which were then stored at 4 °C for use. AuNPs were prepared for characterization by HT7700 transmission electron microscope (TEM, Hitachi), nano ZS zetasizer (Malvern) and lambda-950 UV/vis/NIR spectrophotometer (Perkin Elmer). The cleanliness of the glassware used for the preparation of AuNPs was important for the size and morphological distribution of the AuNPs. All glassware used for the preparation of AuNPs was first ultrasonically cleaned, soaked overnight in freshly prepared aqua regia, then sequentially cleaned with Milli-Q water and dried for later use.

### Cell culture

NRK cells and 293T cells were cultured in Dulbecco’s modified eagle’s medium (DMEM) containing 10% fetal bovine serum (FBS) and 1% penicillin and streptomycin (5% CO_2_, 37 °C). BHK-21 cells were cultured in MEM medium containing 10% fetal bovine serum (FBS) and 1% penicillin and streptomycin (5% CO_2_, 37 °C). The cell viability was analyzed using CCK-8 kits.

### Biological electron microscope

NRK cells were fixed in 4% cold paraformaldehyde and collected by centrifugation, followed by further fixation of the cell blocks with 1% osmotic acid and finally dehydration with various concentrations of ethanol. After embedding with resin, the sample blocks were sectioned at a thickness of 70 nm. This was followed by negative staining with uranyl acetate and lead citrate. Morphological pictures of the cells were obtained by HT7700 TEM.

### Evaluation of lysosomal pH and permeability

NRK cells were seeded in an eight-linked confocal dish at an appropriate density (about 6 × 10^3^/cm^2^) and cultured with AuNPs for 24 h. Discard the culture medium, and then stained with LysoSensor™ Green DND-189 (1:1000) (Thermo, L7535) at 37 °C for 30 min. After washing, cells were observed with LSM710 confocal microscopy (Zeiss) with ×63/1.40 NA oil objective. The excitation wavelength was FITC 488 nm. At the same time, flow cytometry was used to semi-quantitatively analyze the above fluorescence intensity. LysoSensor™ Green DND-189 stained cells were collected. The intracellular fluorescence intensity was detected by Accuri C6 Flow Cytometry (BD Biosciences). Detect channel: FL1, 488 nm, 533 nm/30 nm.

### Immunofluorescent assay

For lysosome-tubulin co-image experiments, cells were fixed with pre-cooled 4% paraformaldehyde for 20 min and punched with immunostaining permeabilization buffer with saponin (P0095, Beyotime) for 5 min at room temperature. After being blocked with 10% goat serum working solution (ZLI-9056, ZSGB-BIO) for 1 h at room temperature, cells were incubated with monoclonal primary antibody of lysosomal-associated membrane protein 1 (LAMP1, ab25630, 1:200) and anti-alpha tubulin antibody (ab18251, 1:1000) at 4 °C overnight. Then, PBS containing Alexa Fluor® 488 labeled goat anti-rabbit IgG (H + L) (1:100) and Alexa Fluor® 594 labeled goat anti-mouse IgG (H + L) (1:100) secondary antibody were added for 1 h at 37 °C. Meanwhile, cells incubated with only the secondary antibody as negative control were used to eliminate the interference of cell autofluorescence. The fluorescence signal was observed by using N-SIM structured-illumination microscope (SIM, Nikon) with a ×100 1.49NA oil objective. Actin was stained with ActinGreen™ 488 ReadyProbes™ Reagent (Thermo, R37110) at 37 °C for 30 min and co-imagined with lysosomes by SIM. For the lysosome swelling assay, NRK cells were transfected with 4 μL/well CellLight Lysosome-RFP (Invitrogen, C10597) for 24 h at 37 °C and then treated with AuNPs for 24 h. After that, lysosome morphology images were recorded by SIM and analyzed with Image J (version 1.8.0, NIH).

### Lysosome mobility assay

To observe real-time lysosomal motility, time-lapse images of lysosomes after AuNPs treatment were recorded. Briefly, NRK cells were transfected with 4 μL/well CellLight Lysosome-RFP for 24 h and treated with AuNPs. After washing with PBS three time to remove residual AuNPs, time-lapse SIM images were captured at 5 s/frame with 561-nm laser over 5 min. Data processing of SIM images was proceeded by NIS-Element (version 4.5).

### AFM

Young’s modulus analysis of cells was conducted using atomic force microscopy (AFM, Agilent-5500) coupled with inverted fluorescence microscopy (Nikon TE2000U). Measurements were performed on cells maintained in culture medium without FBS. Cytoplasmic sites of cells were randomly selected for measurement. The force curves with a frequency of 10 Hz were collected of 5 × 5 points using the cantilever (BudgetSensors) with a force constant. In total, 15 cells for each cell sample were analyzed. The information was calculated to obtain biomechanical parameter.

### Wound-healing assay

A marker pen was prepared to draw horizontal lines on the back of a six-well plate cross the hole evenly for easy positioning. Cell suspension treated or untreated with AuNPs were added to each well (~5 × 10^5^ cells/well), and incubated overnight until confluent. On the second day, a scratch perpendicular to the horizontal line was made using a gun tip. Washed with PBS three times to remove cells that are scribbled off, added serum-free medium, and cultured the cells in 37 °C incubator. Pictures were taken and recorded at the same location at 0, 12, 24, 36, and 48 h.

### Transwell assay

Cells were pre-starved 12 h in serum-free medium to eliminate the influence of serum. The starved cells were digested and resuspended in serum-free medium containing BSA to obtain cell suspension. The lower layer of the transwell chamber is pre-added with 600 μL of 15% serum culture medium, and 200 μL of cell suspension was added slowly into the upper chamber of the transwell and cultured for 8 h at 37 °C. After that, the upper chamber was washed twice with calcium-free PBS and fixed with methanol for 30 min. After drying, the upper chamber was stained with 0.1% crystal violet for 30 min, washed with PBS three times. After gently wiping off the moisture in the upper chamber, the number of migrated cells was counted with 40 times microscope.

### HCoV-OC43 infection experiments

BHK-21 cells were infected with HCoV-OC43 at a multiplicity of infection (MOI) of 0.01. After viral incubation at 33 °C for 2 h, the cells were rinsed with PBS and incubated with culture medium containing different concentrations of AuNPs at 33 °C for 72 h. The copy numbers of viral genes were determined by qRT-PCR assay for the calculation of the inhibition rate of AuNPs against HCoV-OC43.

### Histological and functional analysis of the kidneys

Healthy female BALB/c mice (4-6 weeks) were given AuNPs by tail intravenous injection (100 μL per mouse/ per 3 days) for 21 days. The kidney organs from each mouse were harvested, fixed in 4% fresh paraformaldehyde, blocked into paraffin blocks, and then sliced. Picrosirius red, and PAS staining were then applied to analyze the samples.

### Statistics

Statistical analysis was obtained with GraphPad Prism (version 8, CA). Data were expressed as mean ± SD, and analyzed by two-sided unpaired Student’s *t* test with significance defined as *P < 0.05, **P < 0.01, ***P < 0.001, compared with control. Data were representative of three dependent experiments.

## Supplementary information

Supplementary Materials

Supplementary Videos. 1-4

## Data Availability

The data used in the current study are available from the corresponding authors upon reasonable request.
