# Supplementary material for: Gold nanoparticles combat enveloped RNA virus by affecting organelle dynamics
Source: Signal Transduct Target Ther. 2023 Aug 2;8:285. doi: 10.1038/s41392-023-01562-w (PMC10393956; doi:10.1038/s41392-023-01562-w)
Supplement: Supplementary file 1 — Supplementary Materials [file 41392_2023_1562_MOESM1_ESM.docx]

**Supplemental Materials**

**Gold nanoparticles combat enveloped RNA virus through affecting organelle dynamics**

Fangzhou Li^1#^, Qianqian Huang^1, 2#^, Ziran Zhou^1, 2^, Qiongge Guan^3^, Fei Ye^3^, Baoying Huang^3*^, Weisheng Guo^4*^, Xing-Jie Liang^1,2*^

1 CAS Key Laboratory for Biomedical Effects of Nanomaterials and Nanosafety, CAS Center for Excellence in Nanoscience, National Center for Nanoscience and Technology of China, No. 11, First North Road, Zhongguancun, Beijing, 100190, P.R.China.

2 University of Chinese Academy of Sciences. Beijing 100049, P. R. China.

3 MHC Key Laboratory of Biosafety, National Institute for Viral Disease Control and Prevention, China CDC, Beijing, China.

4 Department of Minimally Invasive Interventional Radiology, the State Key Laboratory of Respiratory Disease, School of Biomedical Engineering & The Second Affiliated Hospital, Guangzhou Medical University, Guangzhou 510260, P. R. China.

# These authors contributed equally to this work.

Correspondence to:

Baoying Huang, [huangby@ivdc.chinacdc.cn](mailto:huangby@ivdc.chinacdc.cn); Weisheng Guo, [tjuguoweisheng@126.com](mailto:tjuguoweisheng@126.com); Xing-Jie Liang, liangxj@nanoctr.cn.

**This file includes:**

Supplementary Table. 1

Supplementary Figure. 1-6

Legend of Supplementary Videos. 1-4

| **Name** | **TEM size (nm)** | **Hydrodynamic size (nm)** | **Z-potential (mV)** |
| --- | --- | --- | --- |
| AuNPs | 49.22±2.0 | 79.6±5.0 | -2.7±0.4 |
| AuNPs (-) | 49.33±3.5 | 78.8±4.7 | -31.3±2.7 |
| AuNPs (+) | 50.23±1.9 | 68.1±4.1 | 25.4±1.4 |

**Supplementary Table. 1 Diameter and zeta potential of the AuNPs used**

**Supplementary Fig. 1**

**Supplementary Fig. 1** Cell viability of NRK cells treated with or without different 1 nM AuNPs.

**Supplementary Fig. 2**

**
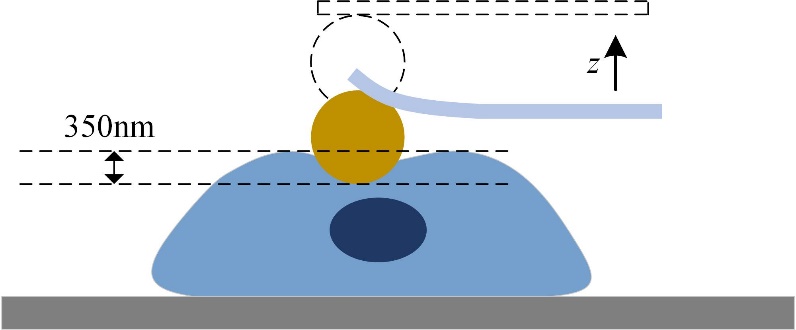
**

**Supplementary Fig. 2** Schematic diagram of AFM indentation method for cell Young’ s module measurement.

**Supplementary Fig. 3**

**a**

**AuNPs (+)**

**Control**


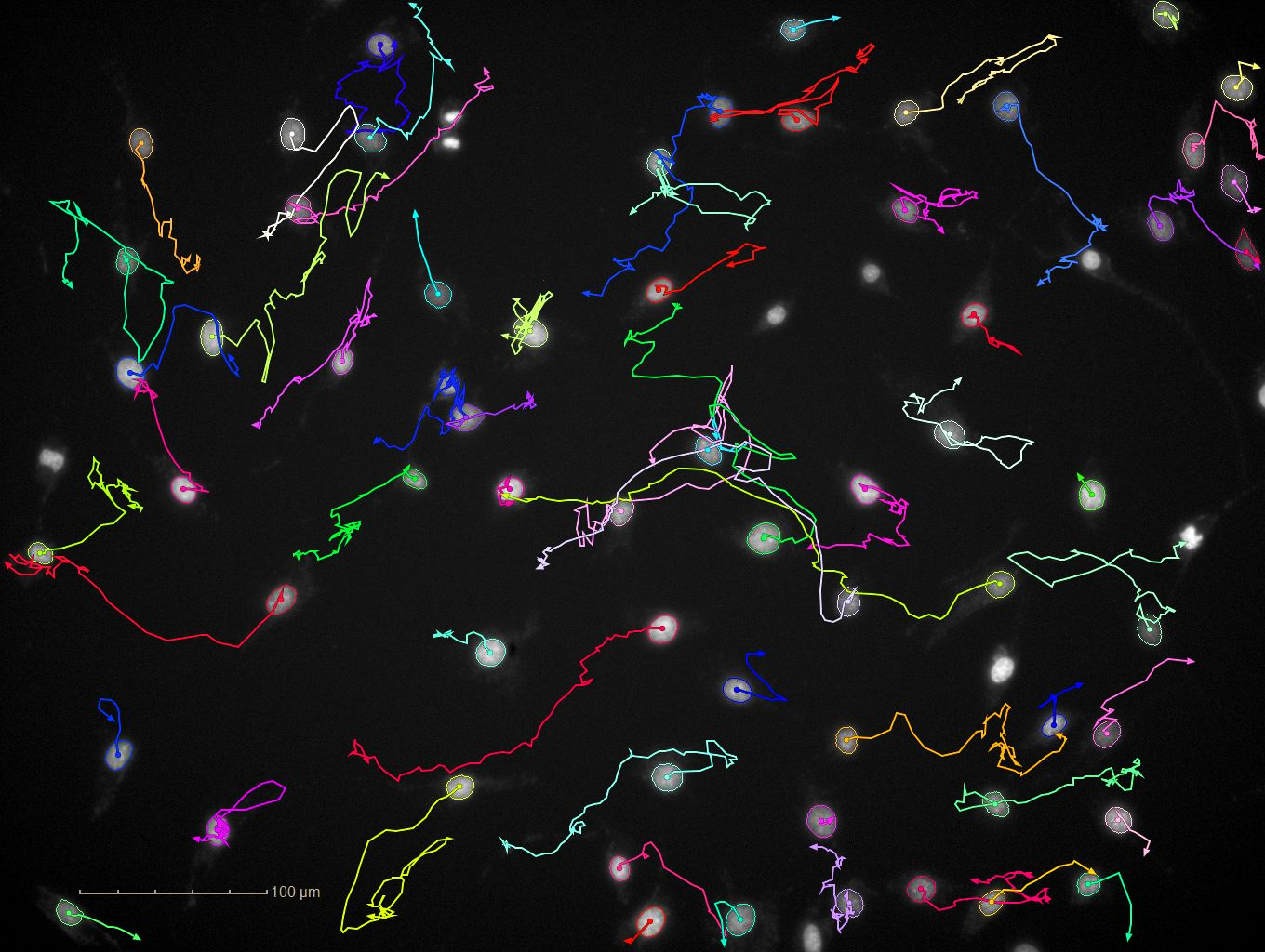

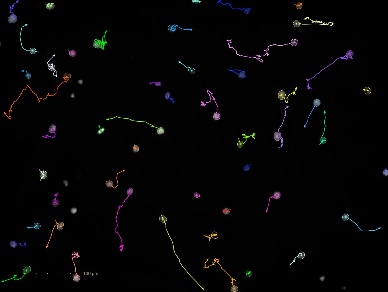

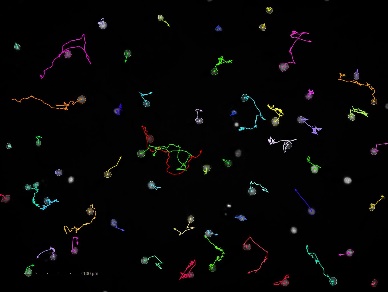


**100 μm**

**Colchicine**


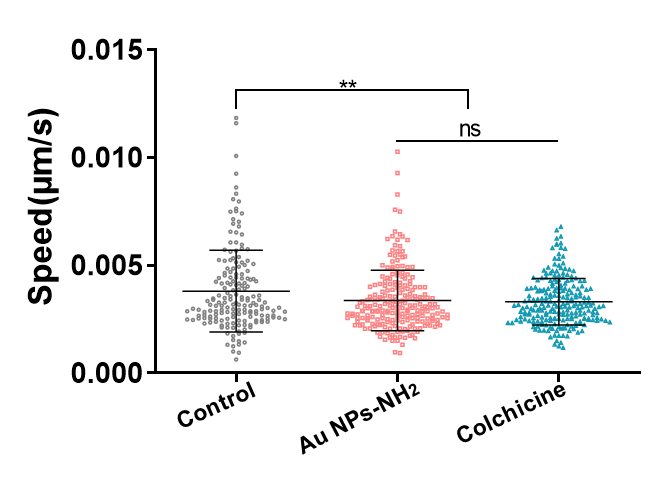


**AuNPs (+)**

**Control**

**Colchicine**


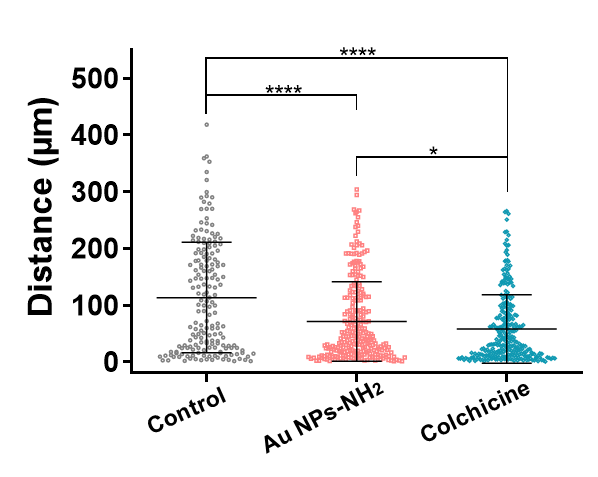


**AuNPs (+)**

**Control**

**Colchicine**

**b**

**c**

**Supplementary Fig. 3** (a) The trajectory of NRK cells measured over 24 h untreated (control) or treated with AuNPs (+) or colchicine (scale bar, 100 μm). (b) Scatter plots of cell migration speed. (c) Cell movement displacement recorded in each group.

**Supplementary Fig. 4**

**Supplementary Fig. 4** Cell viability analysis after treated with different concentrations of AuNPs (+).

**Supplementary Fig. 5**


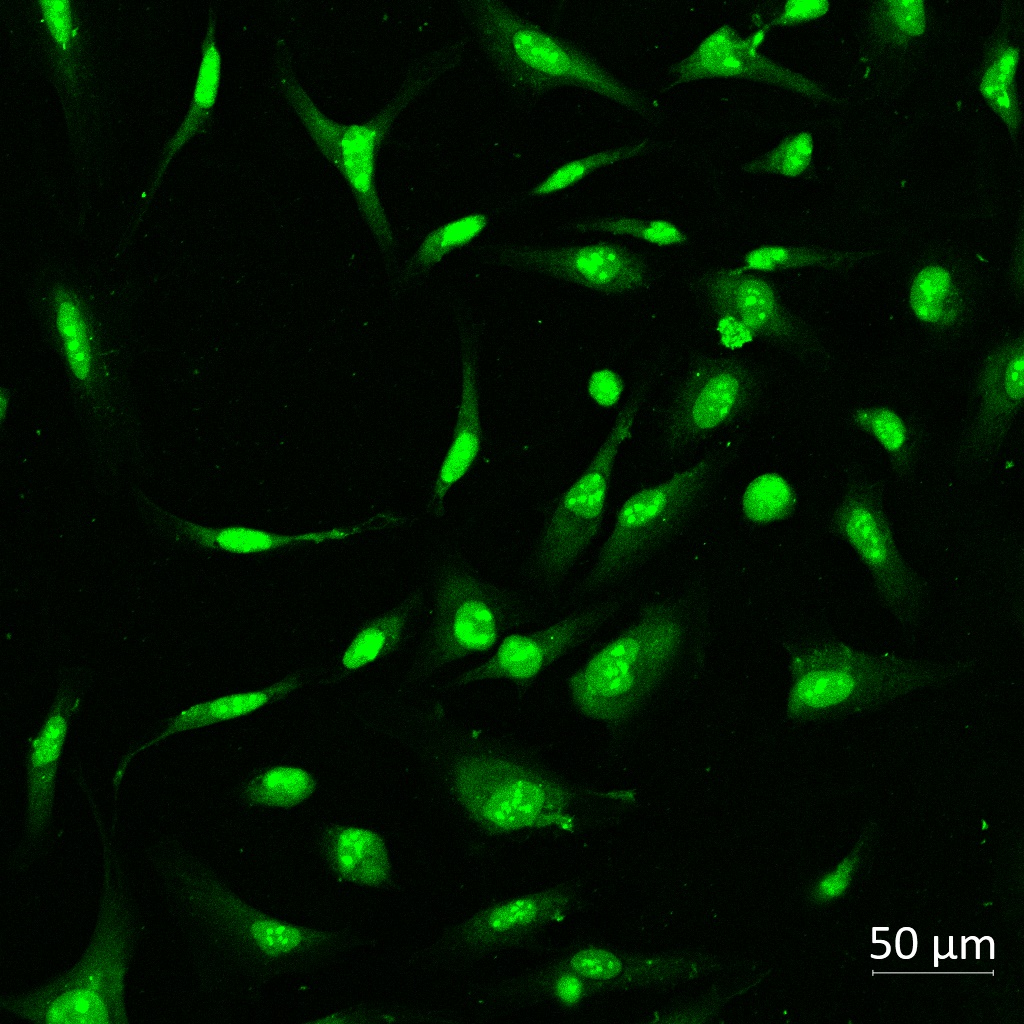

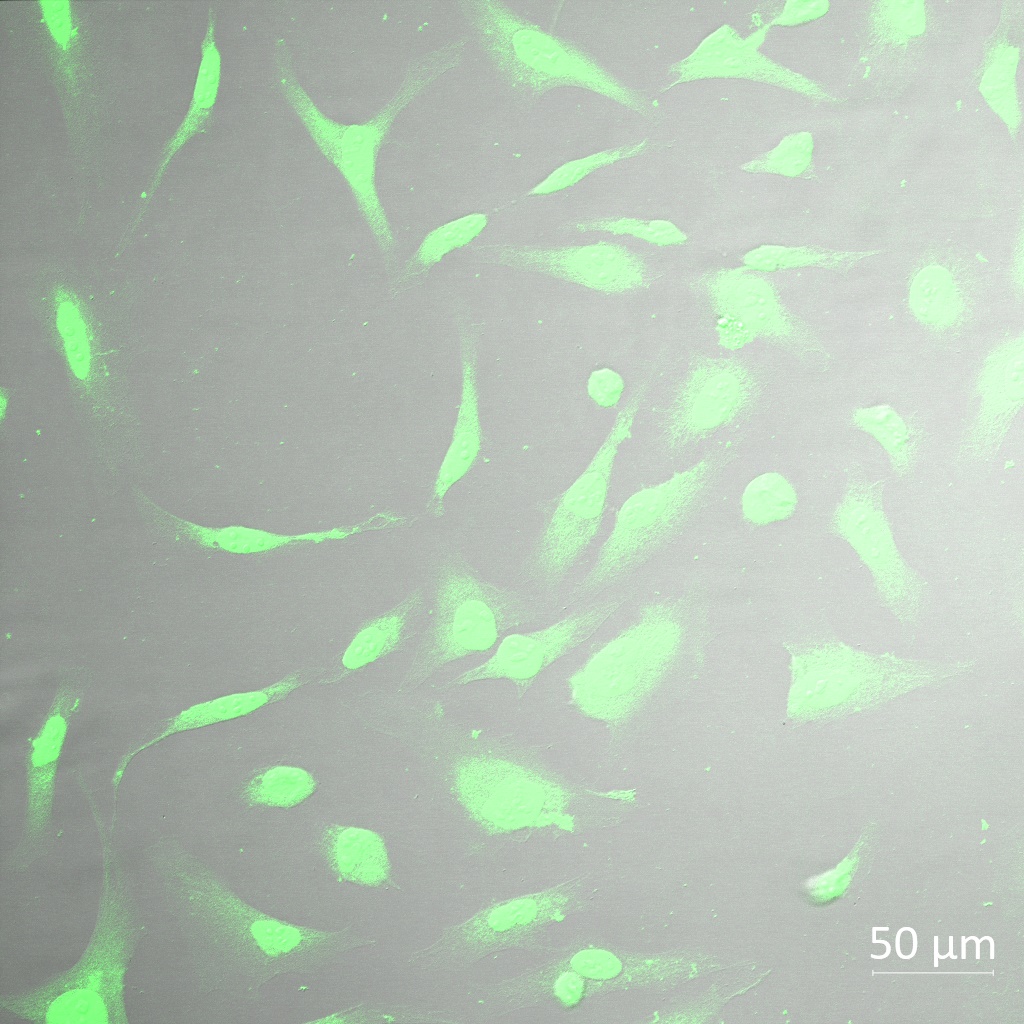

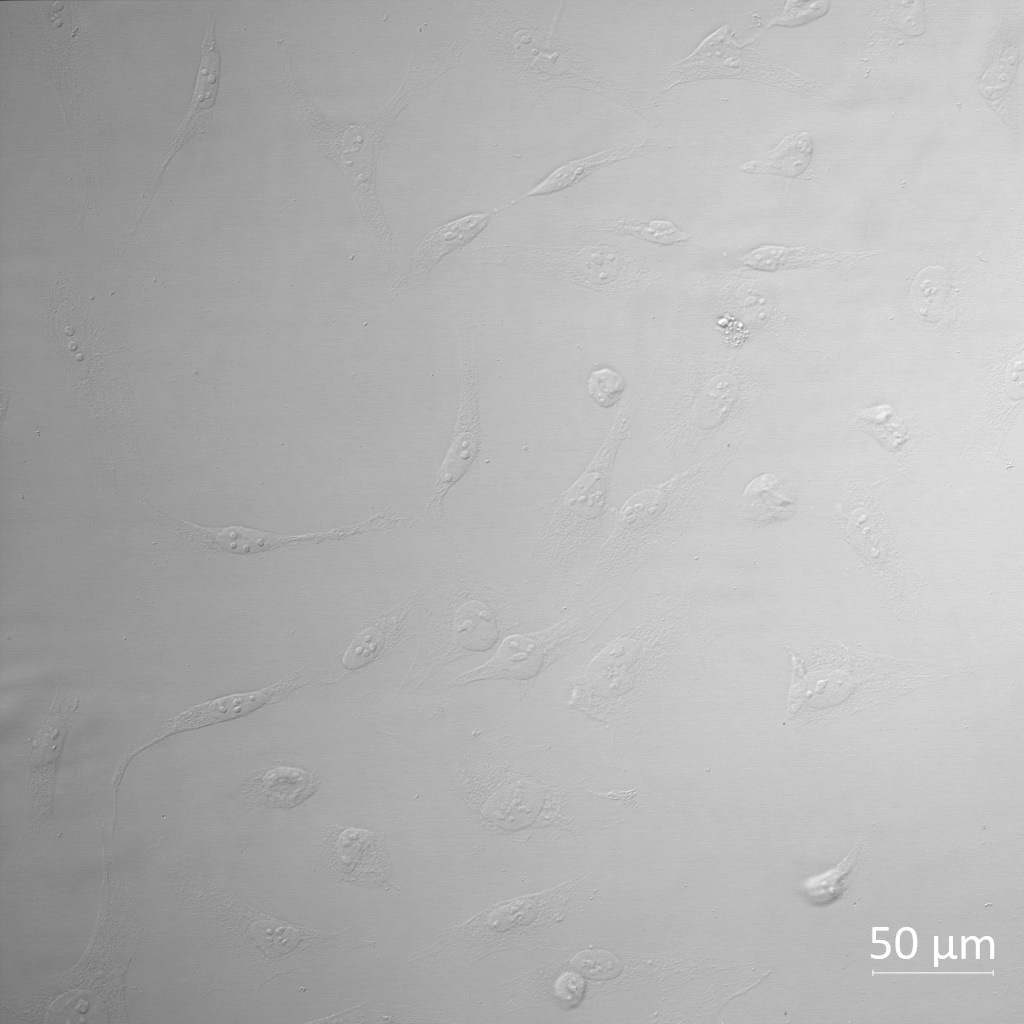

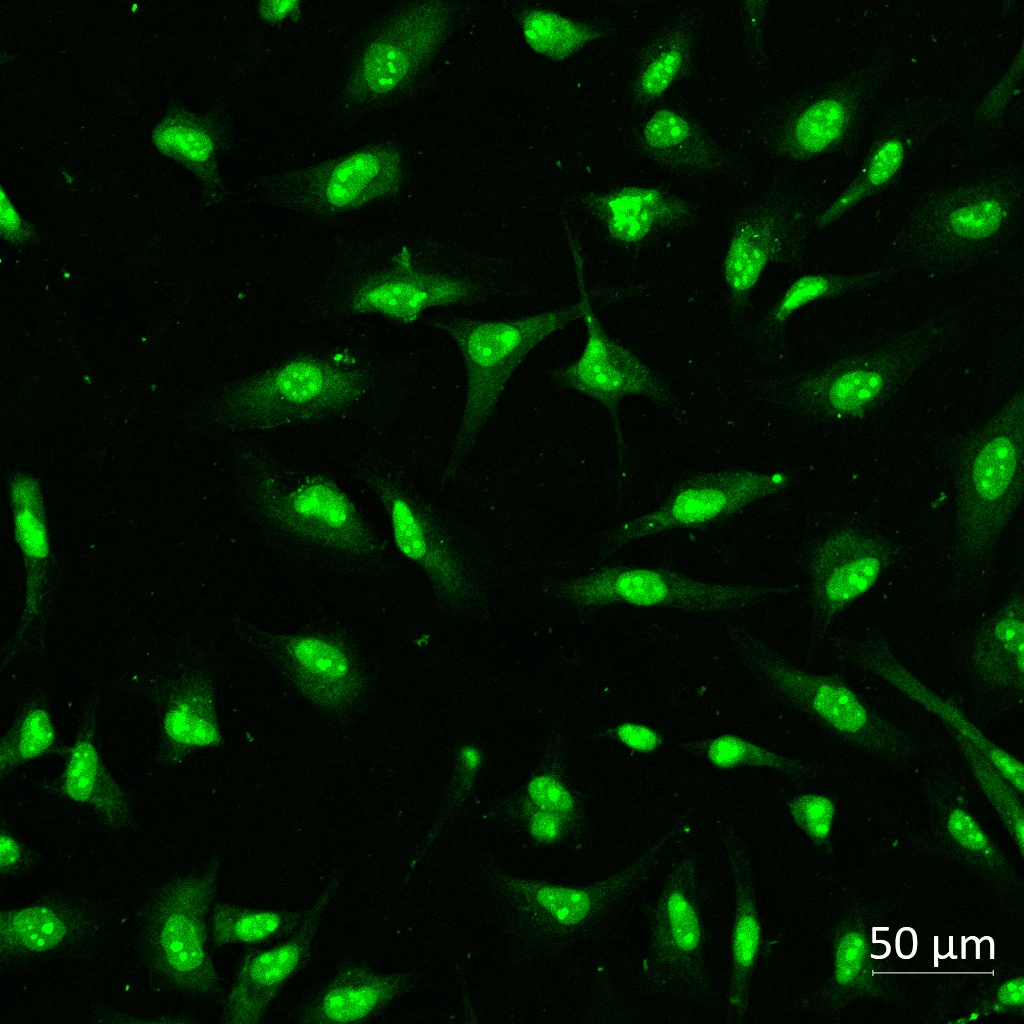

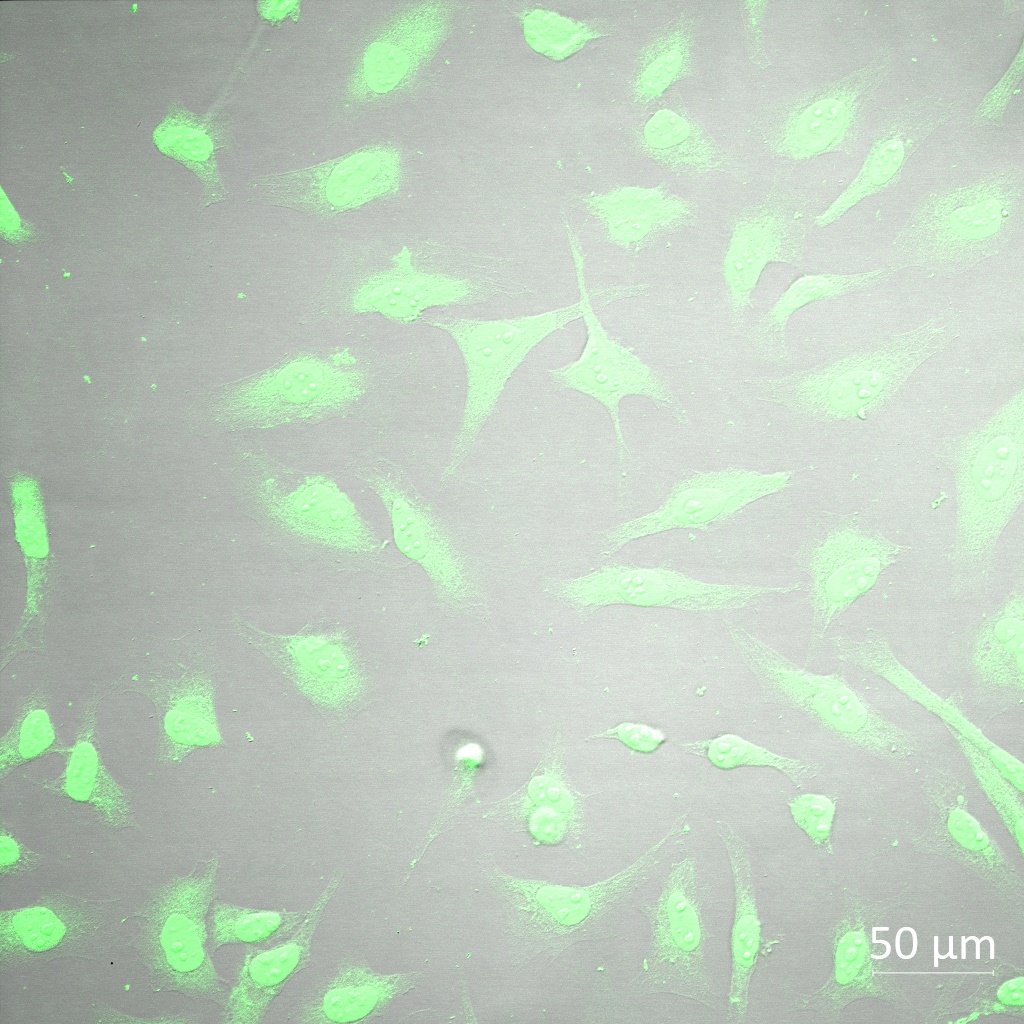

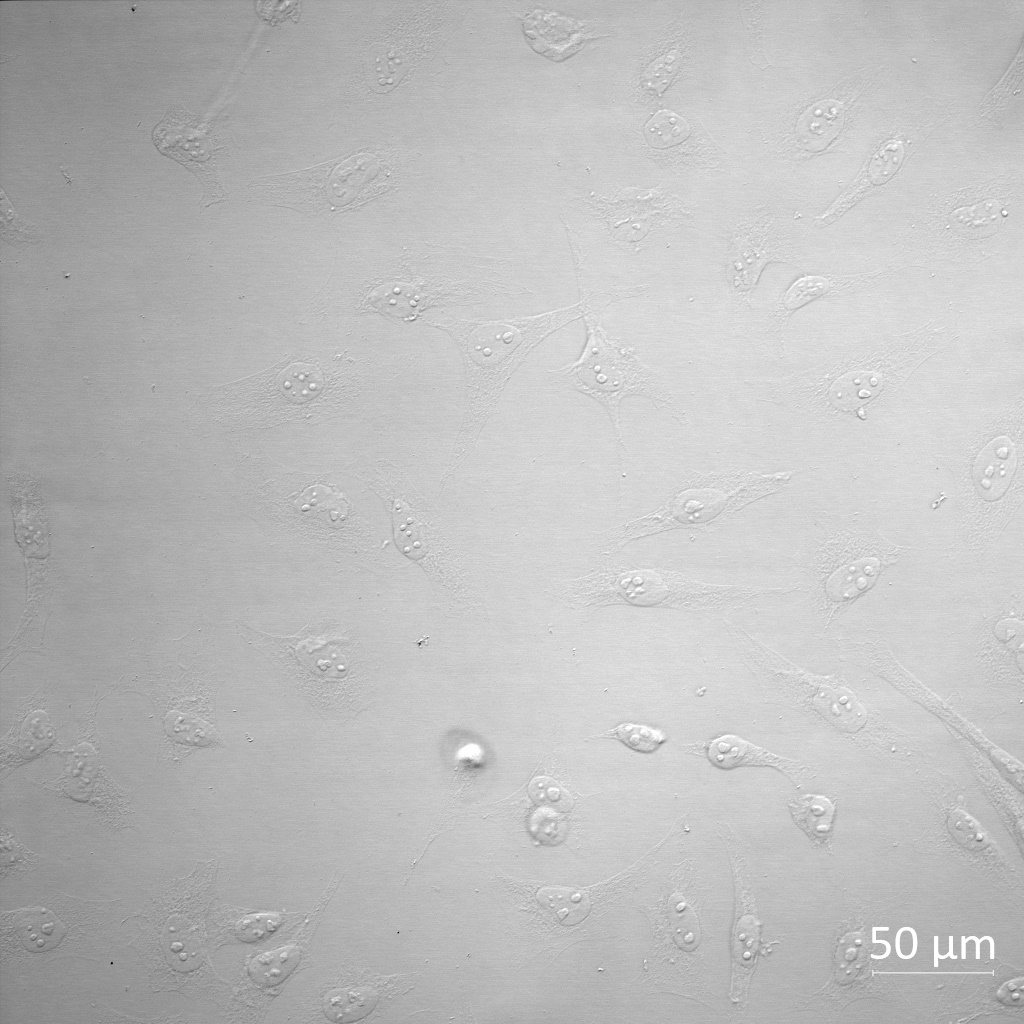

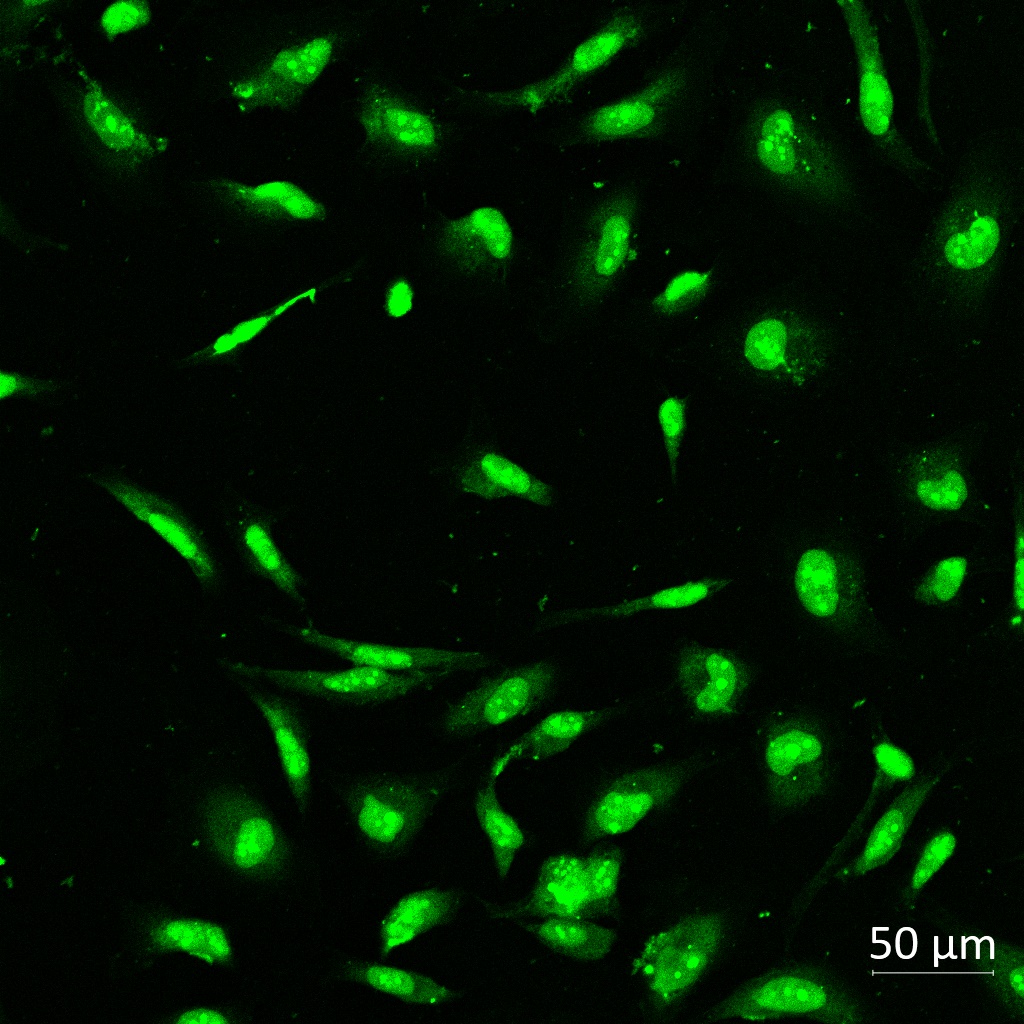

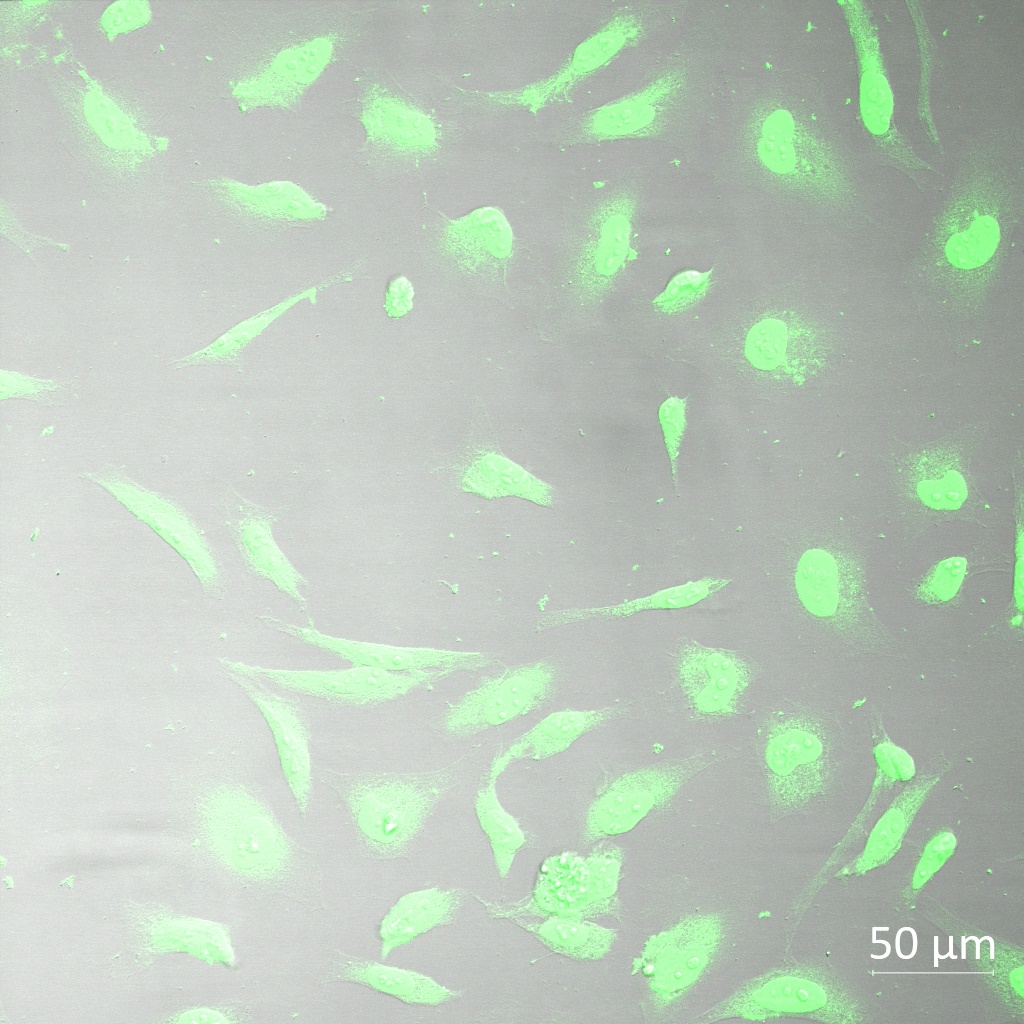

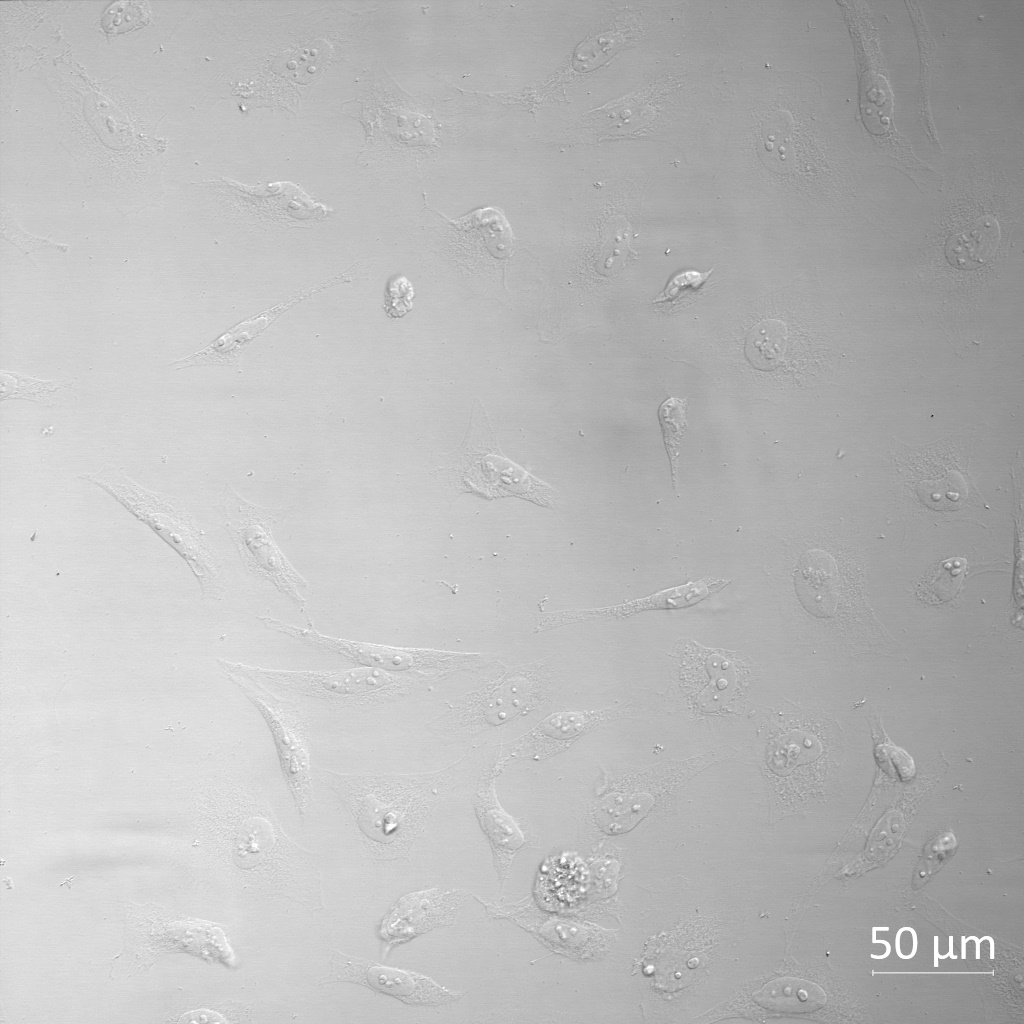

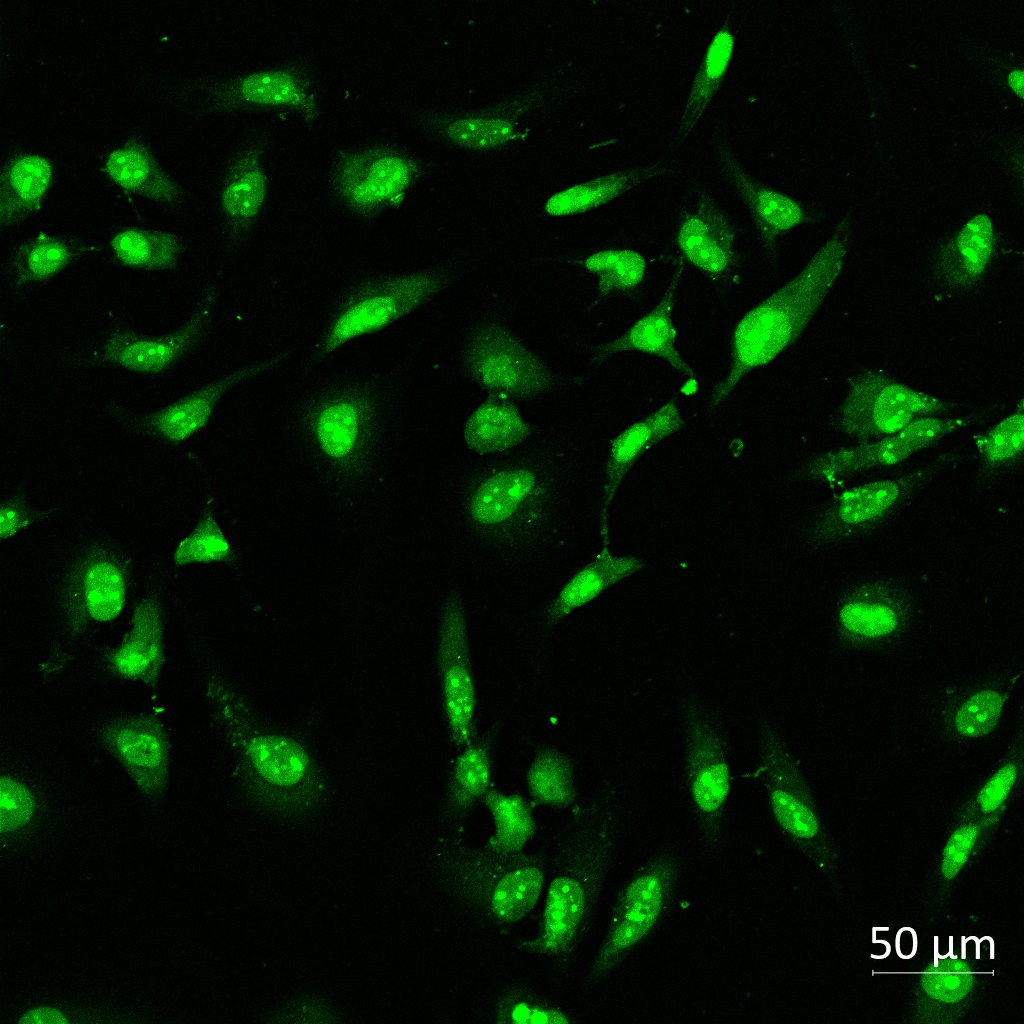

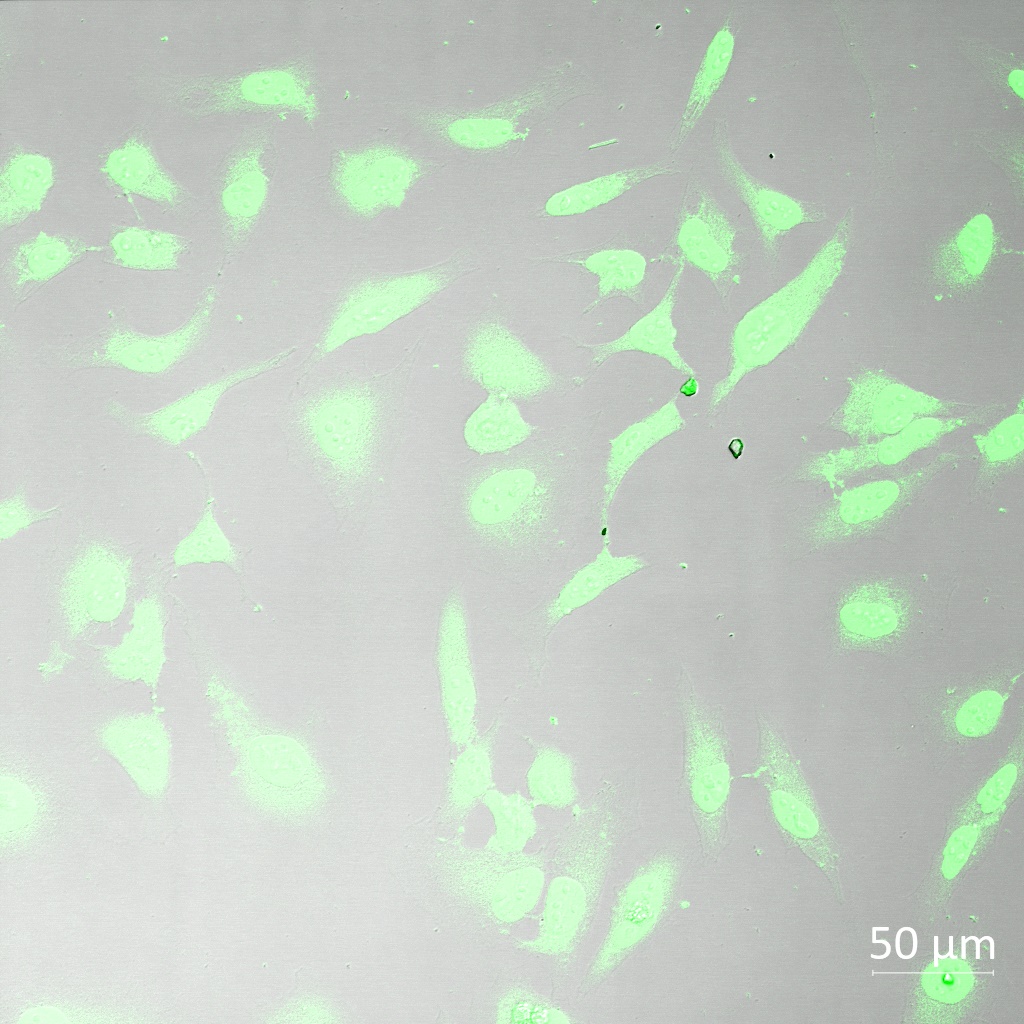

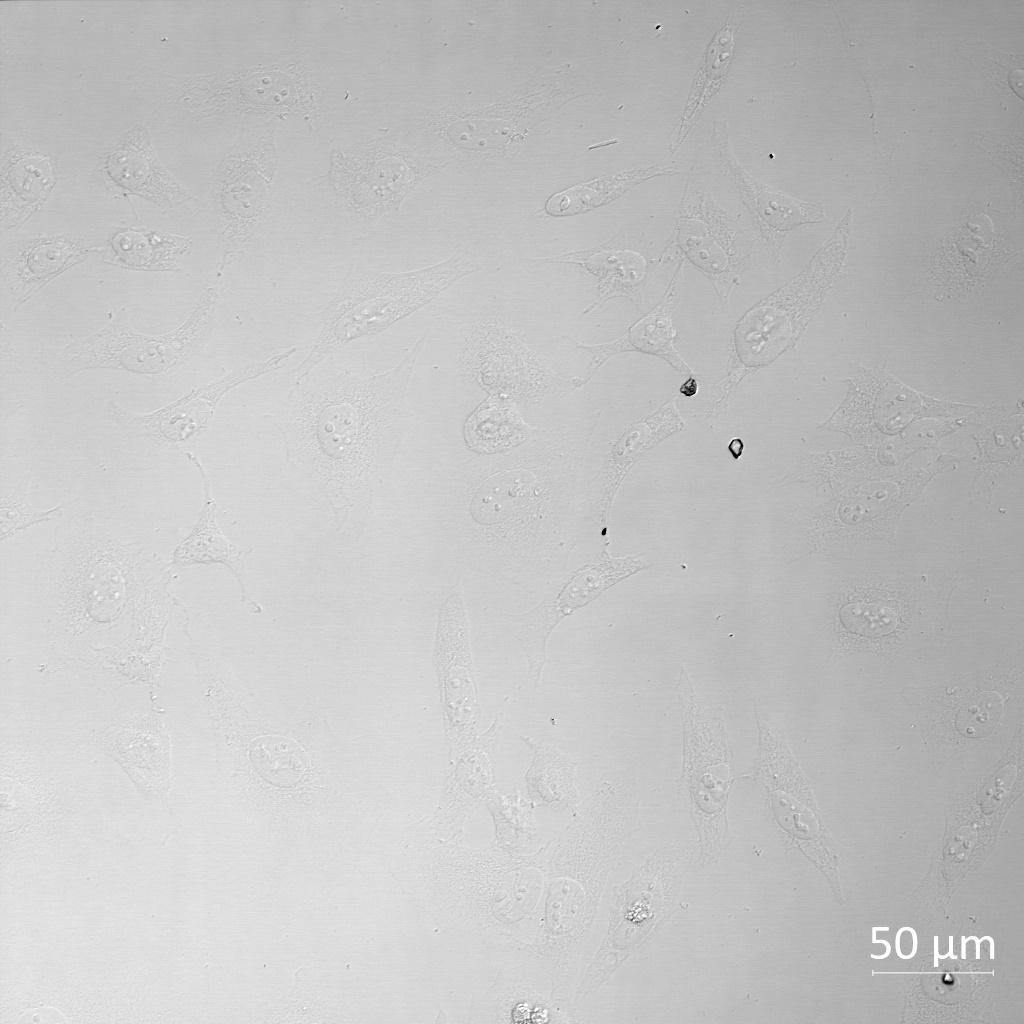

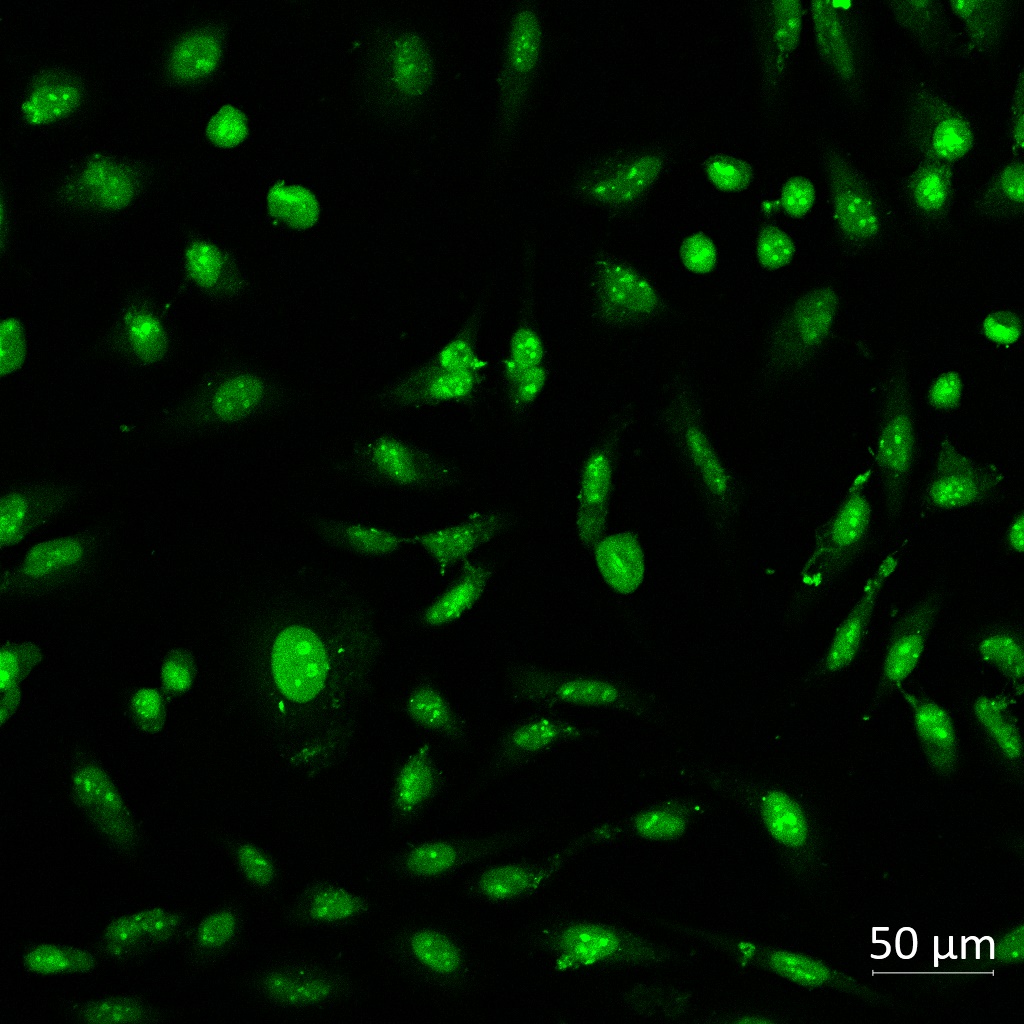

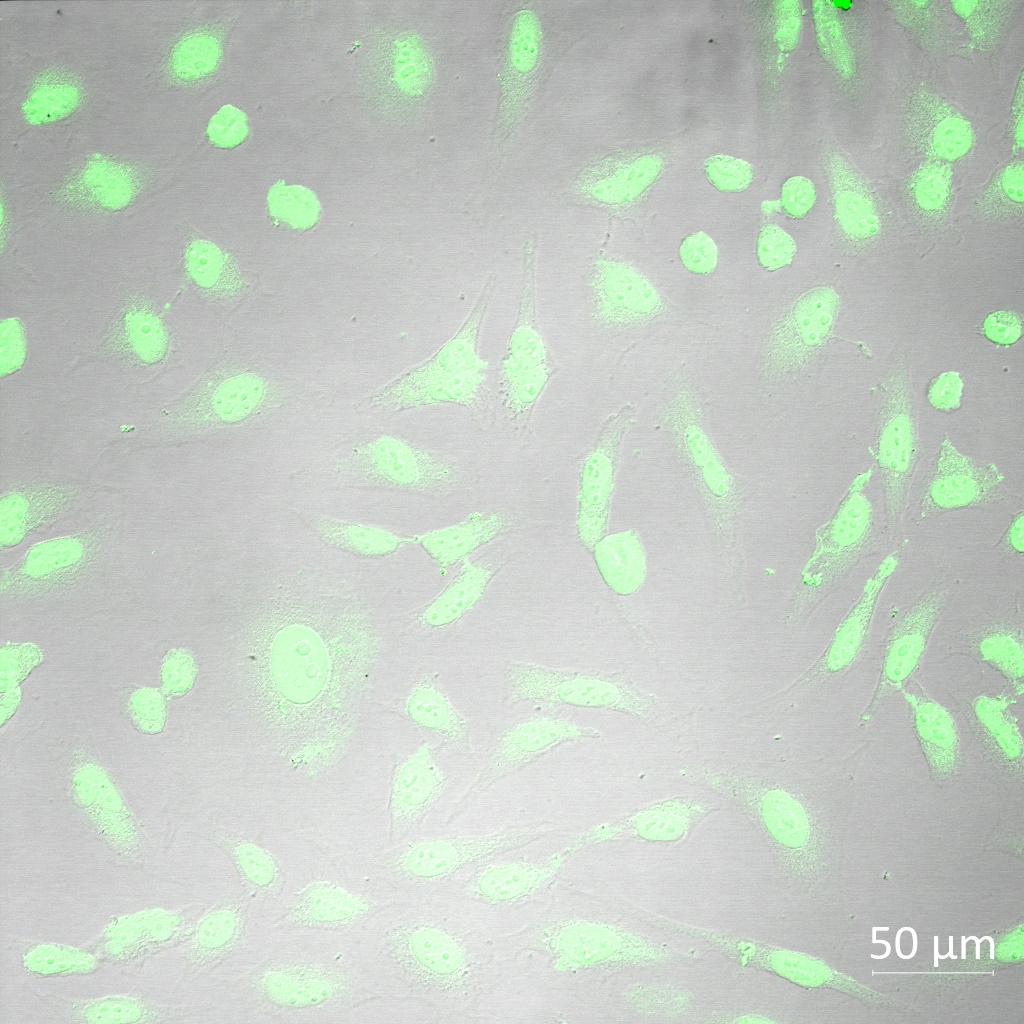

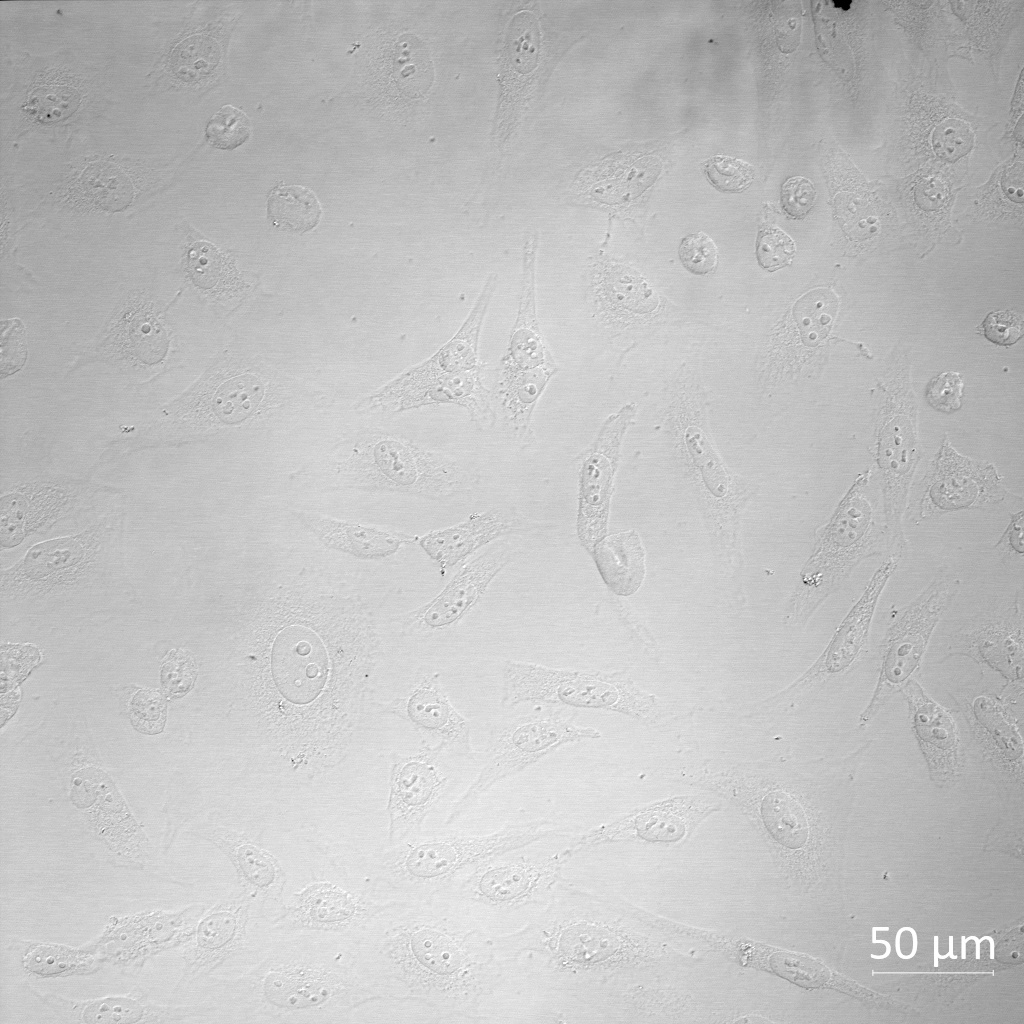


**GFP**

**Merge**

**Bright**

**field**

**PBS**

**0.25 nM**

**0.5 nM**

**1 nM**

**2 nM**

**AuNPs (+)**

**Supplementary Fig. 5** 293T cells were transfected with a triple plasmid system (psPAX2-pMD2.G-pLL3.7) containing the GFP reporter gene to produce lentivirus. After 8 h, the cells were treated with or without AuNPs for 24 h. And then, the cells were prepared for GFP visualization (scale bar, 50 μm).

**Supplementary Fig. 6**

**Supplementary Fig. 6** Body weight change curve of mice treated with or without AuNPs (+).

**Supplementary Videos.** 1-4 Real-time monitoring of lysosomal movement in NRK cells untreated or treated with different AuNPs.
